# Effects of clinical pathways for chronic obstructive pulmonary disease (COPD) on patient, professional and systems outcomes: protocol for a systematic review

**DOI:** 10.1186/s13643-016-0311-8

**Published:** 2016-08-11

**Authors:** Christopher Plishka, Thomas Rotter, Leigh Kinsman, Mohammed Rashaad Hansia, Adegboyega Lawal, Donna Goodridge, Erika Penz, Darcy D. Marciniuk

**Affiliations:** 1 College of Pharmacy and Nutrition, University of Saskatchewan, E3315 Health Sciences Building, Saskatoon, SK S7N 5E5 Canada; 2 University of Tasmania and Tasmanian Health Organisation (North), Launceston, Tasmania Australia; 3 Regina Qu’Appelle Health Region, Regina, Canada; 4 College of Medicine, University of Saskatchewan, Saskatoon, Canada; 5 Respirology, Critical Care and Sleep Medicine, College of Medicine, University of Saskatchewan, Saskatoon, Canada

**Keywords:** Chronic obstructive pulmonary disease, COPD, Clinical pathways, Care pathways, Critical pathways, Integrated care pathways, Care maps

## Abstract

**Background:**

Chronic obstructive pulmonary disease (COPD) is a respiratory syndrome characterized by progressive, partially reversible airway obstruction and lung hyperinflation. COPD has a substantial burden which is seen in both patient quality of life and healthcare costs. One proposed method of minimizing this burden is the implementation of clinical pathways (CPWs). CPWs aim to guide evidence-based practice and improve the interaction between health services. They bring the best available evidence to a range of healthcare professionals by adapting evidence-based clinical guidelines to a local context and detailing the essential steps in the assessment and care of patients.

**Methods:**

The aim of this systematic review is to synthesize existing literature on the effects of CPWs for the treatment or management of COPD. We will screen search hits from search strategies developed for a Cochrane Effective Practice and Organisation of Care (EPOC) systematic review on the use of CPWs in primary care and a Cochrane EPOC review on the use of CPWs in hospitals. These searches were run in a range of databases. Studies will be screened independently by two reviewers. All studies identified by our search strategy will be considered regardless of study design as long as they meet the operational definition for clinical pathways developed by Kinsman et al. (BMC Medicine 8, 2010) and focus on the treatment or management of COPD. All included studies will be evaluated for risk of bias utilizing methodologies set out by the Cochrane collaboration. Data regarding patient, professional and systems outcomes will be extracted from all included studies. Data will be presented in both narrative and tabular form.

**Discussion:**

The systematic review outlined in this protocol aims to identify, assess and synthesise all available evidence on the effects of CPWs regarding the treatment and management of COPD. As a result, this review will provide an evidence base for decision makers regarding the practicality, cost effectiveness, patient benefit and best practices regarding the implementation of CPWs for the care of COPD.

**Electronic supplementary material:**

The online version of this article (doi:10.1186/s13643-016-0311-8) contains supplementary material, which is available to authorized users.

## Background

### Description of the condition

Chronic obstructive pulmonary disease (COPD) is a respiratory syndrome characterized by progressive, partially reversible airway obstruction and lung hyperinflation [1, 2]. This leads to progressive shortness of breath, limitation of daily activities and worsening health-related quality of life, as well as increasingly frequent and severe exacerbations [1]. It is most often caused by exposure to tobacco smoke [3], but is also associated with air pollution and biomass [3] and occupational exposures to dusts and chemicals [3]. The disease is significantly underdiagnosed [4, 5], leading to difficulty in estimating prevalence at both the international and national level. Estimates for worldwide prevalence of COPD range from 4 to 20 % [4]. Similar trends can be seen in Canada where approximately 4 % of Canadians self-report as being diagnosed [5], but estimates based on airflow obstruction suggest a prevalence between 12 and 17 % depending on diagnostic criteria [5]. Data from Europe is less readily available as estimates for Europe in general are out of date [6]. However, statistics from the UK also point to underdiagnoses as 1 million people live with diagnosed COPD while another 2 million are undiagnosed [7].

COPD has significant consequences. It is the fourth leading cause of death in Canada [8] and the third leading cause of death in the USA. In 2009, COPD caused 8 million physician office visits, 1.5 million ED visits, 715,000 hospitalizations and 133,965 deaths in the USA [9]. In 2010, costs for COPD in the US were projected to be $49.9 billion [10]. Exacerbations of COPD (AECOPD) account for most of the morbidity. In Canada alone, the cost of moderate and severe exacerbations has been estimated to be $646–$736 million per year [11]. In the UK, direct costs of COPD are estimated at £800 million [12]. In order to minimize the burden of COPD, high-quality guidelines have been developed [1, 13–16]. These guidelines generally specify disease identification through spirometry, management through a combination of smoking cessation, vaccination, pharmacologic therapy, physical activity, prevention and optimal management of AECOPD [1, 17]. When implemented, these steps have shown substantial increases in patient quality of life, as well as a reduction in healthcare utilization [18].

Although these results are promising, evidence suggests that the creation of guidelines in isolation is inadequate [19–21] as passive dissemination of guidelines rarely results in meaningful changes in practice [14, 22, 23]. Estimates across the healthcare environment suggest that 30–40 % of patients do not receive treatments with proven effectiveness [24], although guideline uptake varies across areas of care [25].

### Description of the intervention

One proposed method of minimizing this gap is the implementation of clinical pathways (CPWs). CPWs, also known as ‘integrated care pathways’, ‘critical pathways’, ‘care plans’ or ‘checklists’, are tools used by health professionals to guide evidence-based practice and improve the interaction between health services. They bring the best available evidence to a range of healthcare professionals by adapting guidelines to a local context and detailing the essential steps in the assessment and care of patients [26, 27]. Evidence suggests that CPWs are commonly implemented and studied in hospitals [28]. However, work analyzing the extent of CPW implementation and evaluation in primary care is still underway [29].

Evidence exists to support the general use of CPWs to change behaviour and improve quality of care [20, 21, 30–32]. Less evidence is available to establish the effectiveness of CPWs for the management of COPD. This is demonstrated in a previous systematic review focused on in-hospital management of AECOPD which utilized a comprehensive search strategy but only identified four studies which met the inclusion criteria [33]. The systematic review described in this protocol follows the preferred reporting items for systematic review and meta-analysis (PRISMA) methodology, as outlined in the PRISMA protocols (PRISMA-P) checklist (see Additional file 1) and will improve the current knowledge base regarding the development and implementation of CPWs for COPD.

## Methods

### Review questions and objectives

The review will address the following question: What are the effects of CPWs for COPD on patient, professional and systems level outcomes. In addition, the secondary objective of the review is to explore factors (e.g. implementation strategies, evidence used in development) that may explain variation in the effectiveness of CPWs for COPD.

For the purpose of this review, usual care will be operationalized as treatment determined at the discretion of the attending healthcare professional. If studies do not state that the control group utilizes some form of standardized care, we will assume the control group utilizes this definition of usual care.

### Criteria for considering studies for this review

The systematic review will include all relevant studies which address the review questions and objectives. We will utilize an electronic search strategy to identify all primary studies reporting on the effectiveness of CPWs for COPD. We will include studies conducted in both primary and acute care settings. In addition, we will include studies focused on COPD maintenance and AECOPD.

### Types of studies

We will include all primary, quantitative studies which utilize the following study designs: randomized controlled trials (RCTs), non-randomized controlled trials (NRCTs), controlled before and after studies (CBA), interrupted time-series (ITS) studies, cohort (longitudinal) studies and pre-post comparisons. We will not include editorial reports or studies which do not collect quantitative data. We will require that all studies include at least one objective pre-intervention/control group measure and one objective post-intervention measure or a measure of change with a corresponding uncertainty measure. Studies which only include a measure of the change or fail to report quantitative results will not be included. We will attempt to contact the study’s author if it is unclear whether the study meets these criteria. We will not exclude studies based on methodological quality; however, study quality will be evaluated using a risk of bias assessment.

### Definition of clinical pathways

In order to ensure we capture all studies utilizing CPWs, regardless of the terminology used, we will rely on the operational definition developed by Kinsman et al. [27] and subsequently refined in the work of Rotter et al. [29] and Lawal et al. [34]. The definition requires that all included studies:

- Utilize a structured multidisciplinary care plan
- Channel the translation of guidelines or evidence into local structures
- Detail the steps in a course of treatment or care in a plan, pathway, algorithm, guideline, protocol or other ‘inventory of actions’ (i.e. the intervention had time frames or criteria-based progression)
- Aim to standardize care for a specific clinical problem, procedure or episode of care in a specific population

### Types of participants

Participants will include patients, care providers and healthcare organizations. All patients with COPD will be included in the review, regardless of COPD severity, diagnostic methods or comorbidities. This will ensure applicability to the care of COPD patients in the ‘real-life’ clinical setting. Regarding care providers, we will consider all health professionals, including but not limited to physicians, nurse practitioners, nurses, physiotherapists, pharmacists, occupational therapists, social workers, dietitians and psychologists. Finally, outcomes regarding healthcare organizations will include all organizations which provide patient care including hospitals and primary care facilities.

### Types of outcome measures

All objectively reported measures which describe a patient, professional or systems level outcome will be included in the review. Patient outcomes are operationalized as outcomes which directly measure the patient’s health or satisfaction after they leave the system. Professional outcomes are operationalized as outcomes which describe how the healthcare professional/staff perceive their work or how they perform tasks but do not measure how efficiently this is done. Finally, systems outcomes are operationalized as outcomes which describe the ability of the system to perform actions using the fewest resources possible. These may influence patients’ experiences but they do not describe the health or satisfaction of the patient following the patient’s visit. Therefore, system outcomes do not include direct measures of health (e.g. mortality rate) and or proxy measures of health (e.g. re-admission rate, infection rate). If studies report pooled data relating to CPWs for COPD in combination with CPWs for other conditions, then outcomes will not be extracted unless a subgroup analysis is presented which shows outcomes for CPWs specific to COPD care.

### Primary outcomes

Objectively measured patient outcomes such as:

- Mortality rate
- Hospital admissions rate
- Emergency department visits
- In-hospital complications rate
- Hospital re-admission rate
- Quality of life
- Patient satisfaction
- Lung function (FEV_1_/FVC and FEV_1_)

Objectively measured professional outcomes such as:

- Guideline adherence
- Employee/clinician satisfaction
- Employee/clinician stress
- Team functioning/collaboration

Objectively defined systems level outcomes such as:

- Length of stay
- Cost

### Search strategies

The review will utilize the search hits resulting from search strategies for a Cochrane Effective Practice and Organisation of Care (EPOC) systematic review on the use of CPWs in primary care and a Cochrane EPOC review update on the use of CPWs in hospitals. These search strategies did not utilize any methodological filters. Search strategies for the review on CPWs in hospitals were run in the following databases:

- MEDLINE (OVID)
- EMBASE (OVID)
- CENTRAL (OVID)
- DARE (OVID)
- Cochrane Database of Systematic Reviews (Wiley)
- WHO international clinical trials registry platform
- ClinicalTrials.gov

Search strategies for the review on CPWs in primary care were run in the following databases:

- MEDLINE (OVID)
- EMBASE (OVID)
- CENTRAL (OVID)
- DARE (OVID)
- Cochrane Database of Systematic Reviews (OVID)

The search focused on CPWs in primary care was run from database inception to 2015. The search focused on CPWs in hospitals was run from 2008 to 2015. Copies of the search strategies for hospitals and primary care translated for the MEDLINE database can be found in Appendices 1 and 2, respectively. Results of these searches will be imported in EndNote X7, and titles, abstracts and keywords will be searched for the following terms:

- COPD
- Chronic obstructive pulmonary disease
- Chronic obstructive airways disease
- Chronic obstructive lung disease
- Emphysema
- Chronic bronchitis

In addition to the search results, we will screen the included and excluded studies lists of the previously published Cochrane systematic review focused on CPWs in hospitals [28] and any other relevant reviews on CPWs identified during screening process. In addition, we will hand search the reference lists for all included studies.

### Screening

All titles and abstracts will be imported into a reference management database and duplicates will be deleted. Two review authors will independently screen all titles and abstracts (CP and LA) to assess which studies meet the inclusion criteria. We will retrieve the full text copies of all potentially relevant articles, and disagreement regarding inclusion will be resolved by a third member of the research team (TR).

### Data extraction

Pairs of two review authors (CP and SF) will independently extract data according to the double data entry method by using a standardized data extraction form developed in Microsoft Access. All data will be extracted directly from included studies. We will refer any disagreements to a third review author (TR). If necessary, we will contact authors of the primary studies for additional information. Areas of data extraction will include:

- Study characteristics: publication year, country, length of follow-up period, urban vs. rural location, inclusion criteria
- Population characteristics (patient): age, gender, number of patients, COPD severity
- Population characteristics (professional): types of healthcare professionals involved, number of healthcare professionals involved in development, healthcare setting
- Intervention characteristics: evidence base, implementation strategy
- Outcomes: patient, professional, systems

### Risk of bias assessment

Two authors (CP and SF) will independently assess the methodological quality of all included studies.

For RCTs, NRCTs and CBA studies, we will use the criteria suggested by the Cochrane EPOC group to assess risk of bias in studies with control groups [35]. These criteria include the following questions:

- Was the allocation sequence adequately generated?
- Was the allocation adequately concealed?
- Were baseline outcome measurements similar?
- Were baseline characteristics similar?
- Were incomplete outcome data adequately addressed?
- Was knowledge of the allocated interventions adequately prevented during the study?
- Was the study adequately protected against contamination?
- Was the study free from selective outcome reporting?
- Was the study free from other risks of bias?

For ITS studies, we will use the criteria suggested by the Cochrane EPOC group to assess risk of bias in ITS studies [35]. These criteria address the following areas:

- Was the intervention independent of other changes?
- Was the shape of the intervention effect pre-specified?
- Was the intervention unlikely to affect data collection?
- Was knowledge of the allocated interventions adequately prevented during the study?
- Were incomplete outcome data adequately addressed?
- Was the study free from selective outcome reporting?
- Was the study free from other risks of bias?

For all types of cohort studies not previously mentioned, we will use the criteria suggested by the Cochrane EPOC group to assess risk of bias in studies with control groups [36]. These criteria include the following questions:

- Is confounding of the effect of intervention unlikely in this study?
- Was selection into the study unrelated to intervention or unrelated to outcome?
- Do start of follow-up and start of intervention coincide for most subjects?
- Is intervention status well defined?
- Was information on intervention status recorded at the time of intervention?
- Was information on intervention status unaffected by knowledge of the outcome or risk of the outcome?
- Were the critical co-interventions balanced across intervention groups?
- Were numbers of switches to other interventions low?
- Was implementation failure minor?
- Are outcome data reasonably complete?
- Was intervention status reasonably complete for those in whom it was sought?
- Are data reasonably complete for other variables in the analysis?
- Was the outcome measure objective?
- Were outcome assessors unaware of the intervention received by study participants?
- Were the methods of outcome assessment comparable across intervention groups?
- Were any systematic errors in measurement of the outcome unrelated to intervention received?
- Is the reported effect estimate unlikely to be selected, on the basis of the results, from multiple outcome measurements within the outcome domain?
- Is the reported effect estimate unlikely to be selected, on the basis of the results, from multiple analyses of the intervention-outcome relationship?
- Is the reported effect estimate unlikely to be selected, on the basis of the results, from different subgroups?

We will refer unresolved disagreements regarding risk of bias to a third author (TR). We will report an appropriate judgement for each criterion on each scale and provide a quote from the study report together with a justification for our judgment in the risk of bias table. Based on individual judgements for each domain, we will provide a summary risk of bias assessment for each study using the method outlined in the Cochrane Handbook which suggests that a study should be rated as low risk of bias if it is plausible bias is unlikely to seriously alter the results, unclear risk of bias if bias raises some doubt about the results and high risk of bias if it is plausible that bias seriously weakens confidence in results [37]. We will not exclude studies from the review based on risk of bias.

### Assessment of heterogeneity

We expect variation due to the fact that we include a range of implementation contexts. However, if there appears to be a body of studies amenable to meta-analysis, then we will inspect graphic representations of pooled results to assess heterogeneity. We will assess statistical heterogeneity both by visual inspection of forest plots and by calculating tests of heterogeneity (Chi^2^ test and *I*^2^ statistic). We will consider an *I*^2^ value greater than 60 % to serve as evidence of substantial heterogeneity of a magnitude where statistical pooling is not appropriate.

### Assessment of reporting biases

We will assess potential reporting biases by visual inspection of funnel plots.

### Subgroup analysis

Subgroup analyses will be conducted for all of the primary and secondary outcomes. We will group studies based on the following categories:

- Country where the study was carried out
- Evidence base used in the development of CPW (e.g. international guidelines, literature review)
- Implementation strategy used in the implementation of the CPW (e.g. passive distribution, face to face training)
- Year of publication
- Context for which the study was focused (e.g. hospitals, primary care)
- Disease stage/severity (stage I/mild COPD, stage II/moderate COPD, stage III/severe COPD and stage IV/very severe COPD)

### Sensitivity analysis

Sensitivity analysis will be carried out to explore the robustness of the results by investigating the effects of including and excluding studies with high risk of bias and studies with missing information. All evidence will be presented in a standardized summary of findings (SoF) table.

### Data analysis

We will undertake meta-analyses if we find more than three studies which report similar outcomes, occur in similar contexts and do not show statistical heterogeneity. Given the fact that there is probably some degree of heterogeneity, it is likely that a random effects model will be employed. However, if studies are sufficiently similar, a fixed effects model will be considered [37].

### Data synthesis

We will record and report details on the number of retrieved references, the number of duplicates, the number of full text papers obtained and the number of included and excluded articles. This information will be reported based on PRISMA guidelines [38]. The reason for exclusion for all articles for which full text was retrieved will be included in the review. A synthesis of risk of bias results will also be presented in tabular form.

Results of meta-analyses will be presented using a forest plot. Outcomes of interest will be synthesized in tabular form and include data for each intervention group, effects estimates, confidence intervals and statistical significance when available. Financial data will be presented in US$ for the same base year and will be adjusted for inflation by using a country-specific price index [39]. Subgroup analyses will be presented in a separate table. In addition, all primary outcomes, meta-analyses and subgroup analyses will be presented in narrative form taking into account the strength of the evidence and following PRISMA guidelines [40].

### Ongoing studies

We will describe identified ongoing studies, where available, detailing the primary author, research question(s), methods and outcome measures together with an estimate of the reporting date.

## Discussion

Overall, the systematic review outlined in this protocol aims to identify, assess and synthesise all available evidence on the effects of CPWs regarding the treatment and management of COPD. As a result, this review will aim to provide an evidence base for decision makers regarding the practicality, cost effectiveness, patient benefit and best practices regarding the implementation of CPWs for the care of COPD.

## Appendix

### Appendix 1: clinical pathways in hospitals search strategy (MEDLINE translation)

1. Critical Pathways/
2. ((clinical or critical) adj2 (pathway? or path)).ti,ab.
3. ((care adj2 algorithm?) or clinical algorithm?).ti,ab.
4. (care adj2 pathway?).ti,ab.
5. (treatment adj3 algorithm?).ti,ab.
6. (structured care or intensive management).ti,ab.
7. (standardi$ adj3 (treatment? or care or patient care or plan$)).ti,ab.
8. (care adj2 (plan? or map or maps or protocol? or algorithm?)).ti,ab.
9. (protocol? adj4 (nursing or treatment or management or directed or guided)).ti,ab.
10. ((local or locally) adj2 adapt$ adj5 guideline?).ti,ab.
11. (treatment model? adj10 standardi$).ti,ab.
12. (standardi$ adj3 (template or templates)).ti,ab.
13. or/1-12 [Pathways]
14. Clinical protocols/
15. Algorithm/and (di.fs. or (treatment or care or patient?).ti. or diagnos$.ti,ab.)
16. Practice Guidelines as Topic/or Guideline Adherence/or Guidelines as topic/
17. ((guideline or guidelines) adj2 (adher$ or implement$)).ti,ab.
18. (guideline? adj4 (compliance or complying)).ti,ab.
19. or/16-18 [PGL or GL Adherence]
20. (adherence or care or compliance or comply$ or implement$ or impact or plan? or standardi?ed or pathway or (treatment adj3 (protocol? or algorithm?))).ti,ab.
21. 19 and 20 [GL]
*Guidelines as topic/or *Practice Guidelines as topic/
*Guideline Adherence/
24. or/22-23 [Focussed MeSH Guideline]
25. Primary health care/or Primary Care Nursing/
26. Family practice/or General Practice/
27. General Practitioners/or Physicians, Family/or Physicians, Primary Care/
28. ((general or family) adj2 (practice? or practitioner? or physician? or doctor?)).ti,ab.
29. (primary adj2 (care or health care or healthcare or medical care or patient care)).ti,ab.
30. (primary care or family medic$ or general practice or family practi$).jn.
31. GP.ti.
32. or/25-31 [Primary Care]
33. Ambulatory Care/or Community medicine/or community health nursing/or community health services/or home care services/or Community mental health services/or Community Pharmacy Services/
34. Ambulatory Care Facilities/or Community Health Centers/
35. (community or communities).ti,ab,hw.
36. (((ambulatory or walk-in or neighbo?rhood or community) adj2 (clinic? or care centre or care centres or care center? or health$ centre or health$ centres or health$ center?)) or public clinic?).ti,ab.
37. ((urban or rural) adj3 health).ti,ab.
38. or/33-37 [Community Care]
39. 13 and 32 [Pathway terms & PC]
40. (and/13,38) not 39 [Pathways & Community-Ambulatory Care]
41. (and/24,32) not (or/39-40) [Focussed GL & PC]
42. (and/24,38) not (or/39-41) [Focussed GL & Community-Ambulatory Care]
43. (21 and (or/32,38)) not (or/39-42) [GL & PC/Amb Care]
44. ((or/14-15) and ((or/26-31,38) or *Primary health care/or *Primary Care Nursing/)) not (or/39-43) [Clinical Protocols/Algorithms Mesh & PC/Community Care-combine with RCT filter only]
45. (randomized controlled trial or controlled clinical trial).pt. or randomized.ab. or placebo.ab. or clinical trials as topic.sh. or randomly.ab. or trial.ti.
46. exp animals/not humans.sh.
47. 45 not 46 [Cochrane RCT Filter 6.4.d Sens/Precision Maximizing]
48. intervention?.ti. or (intervention? adj6 (clinician? or collaborat$ or community or complex or DESIGN$ or doctor? or educational or family doctor? or family physician? or family practitioner? or financial or GP or general practice? or hospital? or impact? or improv$ or individuali?e? or individuali?ing or interdisciplin$ or multicomponent or multi-component or multidisciplin$ or multi-disciplin$ or multifacet$ or multi-facet$ or multimodal$ or multi-modal$ or personali?e? or personali?ing or pharmacies or pharmacist? or pharmacy or physician? or practitioner? or prescrib$ or prescription? or primary care or professional$ or provider? or regulatory or regulatory or tailor$ or target$ or team$ or usual care)).ab.
49. (pre-intervention? or preintervention? or “pre intervention?” or post-intervention? or postintervention? or “post intervention?”).ti,ab. [added 2.4]
50. (hospital$ or patient?).hw. and (study or studies or care or health$ or practitioner? or provider? or physician? or nurse? or nursing or doctor?).ti,hw.
51. demonstration project?.ti,ab.
52. (pre-post or “pre test$” or pretest$ or posttest$ or “post test$” or (pre adj5 post)).ti,ab.
53. (pre-workshop or post-workshop or (before adj3 workshop) or (after adj3 workshop)).ti,ab.
54. trial.ti. or ((study adj3 aim?) or “our study”).ab.
55. (before adj10 (after or during)).ti,ab.
56. (“quasi-experiment$” or quasiexperiment$ or “quasi random$” or quasirandom$ or “quasi control$” or quasicontrol$ or ((quasi$ or experimental) adj3 (method$ or study or trial or design$))).ti,ab,hw.
57. (“time series” adj2 interrupt$).ti,ab,hw.
58. (time points adj3 (over or multiple or three or four or five or six or seven or eight or nine or ten or eleven or twelve or month$ or hour? or day? or “more than”)).ab.
59. pilot.ti.
60. Pilot projects/
61. (clinical trial or controlled clinical trial or multicenter study).pt.
62. (multicentre or multicenter or multi-centre or multi-center).ti.
63. random$.ti,ab. or controlled.ti.
64. (control adj3 (area or cohort? or compare? or condition or design or group? or intervention? or participant? or study)).ab. not (controlled clinical trial or randomized controlled trial).pt.
65. evaluation studies as topic/or prospective studies/or retrospective studies/[Added Jan 2013]
66. (utili?ation or programme or programmes).ti. [Added Jan 2013]
67. (during adj5 period).ti,ab. [Added Jan 2013]
68. ((strategy or strategies) adj2 (improv$ or education$)).ti,ab. [Added Jan 2013]
69. “comment on”.cm. or review.pt. or (review not “peer review$”).ti. or randomized controlled trial.pt. [Changed Jan 2013]
70. (rat or rats or cow or cows or chicken? or horse or horses or mice or mouse or bovine or animal?).ti.
71. exp animals/not humans.sh.
72. (or/48-68) not (or/69-71) [EPOC Methods Filter 2.5-added Evaluation Studies line forward--Jan 20130 Medline]
73. (or/39-44) and 47 [RCT Results]
74. (39 and 72) not 73 [EPOC Filter Results Set 1 : Pathways & PC]
75. (40 and 72) not (or/73-74) [EPOC Filter Set 2: Pathways & Community-Ambulatory Care]
76. (41 and 72) not (or/73-75) [EPOC Filter Set 3: Focussed GL & PC]
77. (42 and 72) not (or/73-76) [EPOC Filter Set 4: Focussed GL & Ambultory]
78. (43 and 72) not (or/73-77) [EPOC Filter Set 5: GL & PC/Amb care]
79. or/74-78 [EPOC Filter Results]
80. 73 or 79

### Appendix 2: clinical pathways in primary care search strategy (MEDLINE translation)

1. Evoked Potentials, Somatosensory/(11114)
2. Critical Pathways/(4597)
3. ((clinical or critical) adj2 (pathway? or path)).ti,ab. (6415)
4. (care adj2 algorithm?) or clinical algorithm?).ti,ab. (1016)
5. (care adj2 pathway?).ti,ab. (2078)
6. (treatment adj3 algorithm?).ti,ab. (4585)
7. (structured care or intensive management).ti,ab. (743)
8. (standardi$ adj3 (treatment? or care or patient care or plan$)).ti,ab. (4945)
9. (care adj2 (plan? or map or maps or protocol? or algorithm?)).ti,ab. (9151)
10. (protocol? adj4 (nursing or treatment or management or directed or guided)).ti,ab. (21924)
11. ((local or locally) adj2 adapt$ adj5 guideline?).ti,ab. (70)
12. (treatment model? adj10 standardi$).ti,ab. (8)
13. (standardi$ adj3 (template or templates)).ti,ab. (210)
14. or/2-13 [Pathways] (51371)
15. Clinical protocols/(19906)
16. Algorithm/and (di.fs. or (treatment or care or patient?).ti. or diagnos$.ti,ab.) (38867)
17. Practice Guidelines as Topic/or Guideline Adherence/or Guidelines as topic/(122045)
18. ((guideline or guidelines) adj2 (adher$ or implement$)).ti,ab. (4692)
19. (guideline? adj4 (compliance or complying)).ti,ab. (2523)
20. or/17-19 [PGL or GL Adherence] (124948)
21. (adherence or care or compliance or comply$ or implement$ or impact or plan? or standardi?ed or pathway or (treatment adj3 (protocol? or algorithm?))).ti,ab. (2441299)
22. 20 and 21 [GL] (47061)
*Guidelines as topic/or *Practice Guidelines as topic/(34513)
*Guideline Adherence/(9488)
25. or/23-24 [Focussed MeSH Guideline] (41286)
26. Primary health care/or Primary Care Nursing/(54165)
27. Family practice/or General Practice/(63815)
28. General Practitioners/or Physicians, Family/or Physicians, Primary Care/(17793)
29. ((general or family) adj2 (practice? or practitioner? or physician? or doctor?)).ti,ab. (91238)
30. (primary adj2 (care or health care or healthcare or medical care or patient care)).ti,ab. (87195)
31. (primary care or family medic$ or general practice or family practi$).jn. (8099)
32. GP.ti. (3130)
33. or/26-32 [Primary Care] (213442)
34. Ambulatory Care/or Community medicine/or community health nursing/or community health services/or home care services/or Community mental health services/or Community Pharmacy Services/(121247)
35. Ambulatory Care Facilities/or Community Health Centers/(17729)
36. (community or communities).ti,ab,hw. (390492)
37. (((ambulatory or walk-in or neighbo?rhood or community) adj2 (clinic? or care centre or care centres or care center? or health$ centre or health$ centres or health$ center?)) or public clinic?).ti,ab. (9467)
38. ((urban or rural) adj3 health).ti,ab. (10234)
39. or/34-38 [Community Care] (460698)
40. 14 and 33 [Pathway terms & PC] (2674)
41. (and/14,39) not 40 [Pathways & Community-Ambulatory Care] (3006)
42. (and/25,33) not (or/40-41) [Focussed GL & PC] (3031)
43. (and/25,39) not (or/40-42) [Focussed GL & Community-Ambulatory Care] (1998)
44. (22 and (or/33,39)) not (or/40-43) [GL & PC/Amb Care] (6172)
45. ((or/15-16) and ((or/27-32,39) or *Primary health care/or *Primary Care Nursing/)) not (or/40-44) [Clinical Protocols/Algorithms Mesh & PC/Community Care-combine with RCT filter only] (3119)
46. (randomized controlled trial or controlled clinical trial).pt. or randomized.ab. or placebo.ab. or clinical trials as topic.sh. or randomly.ab. or trial.ti. (906690)
47. exp animals/not humans.sh. (3947170)
48. 46 not 47 [Cochrane RCT Filter 6.4.d Sens/Precision Maximizing] (836655)
49. intervention?.ti. or (intervention? adj6 (clinician? or collaborat$ or community or complex or DESIGN$ or doctor? or educational or family doctor? or family physician? or family practitioner? or financial or GP or general practice? or hospital? or impact? or improv$ or individuali?e? or individuali?ing or interdisciplin$ or multicomponent or multi-component or multidisciplin$ or multi-disciplin$ or multifacet$ or multi-facet$ or multimodal$ or multi-modal$ or personali?e? or personali?ing or pharmacies or pharmacist? or pharmacy or physician? or practitioner? or prescrib$ or prescription? or primary care or professional$ or provider? or regulatory or regulatory or tailor$ or target$ or team$ or usual care)).ab. (163136)
50. (pre-intervention? or preintervention? or “pre intervention?” or post-intervention? or postintervention? or “post intervention?”).ti,ab. [added 2.4] (10329)
51. (hospital$ or patient?).hw. and (study or studies or care or health$ or practitioner? or provider? or physician? or nurse? or nursing or doctor?).ti,hw. (720195)
52. demonstration project?.ti,ab. (1958)
53. (pre-post or “pre test$” or pretest$ or posttest$ or “post test$” or (pre adj5 post)).ti,ab. (66138)
54. (pre-workshop or post-workshop or (before adj3 workshop) or (after adj3 workshop)).ti,ab. (617)
55. trial.ti. or ((study adj3 aim?) or “our study”).ab. (634701)
56. (before adj10 (after or during)).ti,ab. (358281)
57. (“quasi-experiment$” or quasiexperiment$ or “quasi random$” or quasirandom$ or “quasi control$” or quasicontrol$ or ((quasi$ or experimental) adj3 (method$ or study or trial or design$))).ti,ab,hw. (101852)
58. (“time series” adj2 interrupt$).ti,ab,hw. (1067)
59. (time points adj3 (over or multiple or three or four or five or six or seven or eight or nine or ten or eleven or twelve or month$ or hour? or day? or “more than”)).ab. (9207)
60. pilot.ti. (39999)
61. Pilot projects/(82514)
62. (clinical trial or controlled clinical trial or multicenter study).pt. (627959)
63. (multicentre or multicenter or multi-centre or multi-center).ti. (29139)
64. random$.ti,ab. or controlled.ti. (759522)
65. (control adj3 (area or cohort? or compare? or condition or design or group? or intervention? or participant? or study)).ab. not (controlled clinical trial or randomized controlled trial).pt. (412029)
66. evaluation studies as topic/or prospective studies/or retrospective studies/[Added Jan 2013] (958573)
67. (utili?ation or programme or programmes).ti. [Added Jan 2013] (54870)
68. (during adj5 period).ti,ab. [Added Jan 2013] (300309)
69. ((strategy or strategies) adj2 (improv$ or education$)).ti,ab. [Added Jan 2013] (18447)
70. “comment on”.cm. or review.pt. or (review not “peer review$”).ti. or randomized controlled trial.pt. [Changed Jan 2013] (2926583)
71. (rat or rats or cow or cows or chicken? or horse or horses or mice or mouse or bovine or animal?).ti. (1343855)
72. exp animals/not humans.sh. (3947170)
73. (or/49-69) not (or/70-72) [EPOC Methods Filter 2.5-added Evaluation Studies line forward--Jan 20130 Medline] (2833476)
74. (or/40-45) and 48 [RCT Results] (2611)
75. (40 and 73) not 74 [EPOC Filter Results Set 1: Pathways & PC] (1116)
76. (41 and 73) not (or/74-75) [EPOC Filter Set 2: Pathways & Community-Ambulatory Care] (1416)
77. (42 and 73) not (or/74-76) [EPOC Filter Set 3: Focussed GL & PC] (985)
78. (43 and 73) not (or/74-77) [EPOC Filter Set 4: Focussed GL & Ambultory] (739)
79. (44 and 73) not (or/74-78) [EPOC Filter Set 5: GL & PC/Amb care] (2295)
80. or/75-79 [EPOC Filter Results] (6551)
81. 74 or 80

## Abbreviations

AECOPD, exacerbations of chronic obstructive pulmonary disease; CBA, controlled before and after study; COPD, chronic obstructive pulmonary disease; CPW, clinical pathway; FEV_1_, forced expiratory volume at 1 s; FEV_1_/FVC, forced expiratory volume at 1 s/forced vital capacity; ITS, interrupted time series; NRCT, non-randomized controlled trial; RCT, randomized controlled trial; SoF, summary of findings

## Additional files

Additional file 1: PRISMA-P checklist. (DOCX 28 kb)
